# Imaging as an early biomarker to predict sensitivity to everolimus for progressive NF2-related vestibular schwannoma

**DOI:** 10.1007/s11060-024-04596-4

**Published:** 2024-02-19

**Authors:** Phioanh Leia Nghiemphu, Jeremie Vitte, Eva Dombi, Thien Nguyen, Naveed Wagle, Akira Ishiyama, Ali R. Sepahdari, David Cachia, Brigitte C. Widemann, Derald E. Brackmann, Joni K. Doherty, Michel Kalamarides, Marco Giovannini

**Affiliations:** 1 Department of Neurology, UCLA Neuro‑Oncology Program, David Geffen School of Medicine and Jonsson Comprehensive Cancer Center (JCCC), University of California, Los Angeles, Los Angeles, CA USA; 2 Department of Head and Neck Surgery, David Geffen School of Medicine and Jonsson Comprehensive Cancer Center (JCCC), University of California, Los Angeles, 675 Charles E Young Dr. S, MRL 2240, Los Angeles, CA 90095-7286 USA; 3 Pediatric Oncology Branch, National Cancer Institute, Bethesda, MD USA; 4 Department of Medicine, Division of Medical Oncology, Norris Cancer Center, University of Southern California, Los Angeles, CA USA; 5 Department of Radiology, David Geffen School of Medicine, University of California, Los Angeles, Los Angeles, CA USA; 6 Department of Neurosurgery, Division of Neuro-oncology, Medical University of South Carolina, Charleston, SC USA; 7 Department of Otolaryngology and Neurotology, House Clinic and Research Institute, Los Angeles, CA USA; 8 Center for Neural Tumor Research, House Research Institute, Los Angeles, CA USA; 9 Department of Neurosurgery, Hôpital Pitié-Salpêtrière, APHP, Sorbonne Université, Paris, France; 10 Present Address: Department of Pediatrics, Division of Pediatric Hematology, Oncology, Stem Cell Transplant and Regenerative Medicine, Stanford University, Palo Alto, CA USA; 11 Present Address: Department of Translational Neurosciences, Saint John’s Cancer Institute at Providence Saint John’s Health Center, Santa Monica, CA USA; 12 Present Address: Diagnostic Neuroradiology, Scripps Clinic Medical Group, La Jolla, CA USA; 13 Present Address: Department of Medicine, Division of Hematology/Oncology, University of Massachusetts, Worcester, MA USA; 14 Present Address: Department of Otolaryngology - Head and Neck Surgery, University of Southern California, Los Angeles, CA USA

**Keywords:** Vestibular schwannoma, NF2, Everolimus, Mammalian target of rapamycin (mTOR) inhibitors, Clinical trial, Quantitative imaging biomarkers

## Abstract

**Purpose:**

*NF2*-related schwannomatosis (NF2) is characterized by bilateral vestibular schwannomas (VS) often causing hearing and neurologic deficits, with currently no FDA-approved drug treatment. Pre-clinical studies highlighted the potential of mTORC1 inhibition in delaying schwannoma progression. We conducted a prospective open-label, phase II study of everolimus for progressive VS in NF2 patients and investigated imaging as a potential biomarker predicting effects on growth trajectory.

**Methods:**

The trial enrolled 12 NF2 patients with progressive VS. Participants received oral everolimus daily for 52 weeks. Brain imaging was obtained quarterly. As primary endpoint, radiographic response (RR) was defined as ≥ 20% decrease in target VS volume. Secondary endpoints included other tumors RR, hearing outcomes, drug safety and quality of life (QOL).

**Results:**

Eight participants completed the trial and four discontinued the drug early due to significant volumetric VS progression. After 52 weeks of treatment, the median annual VS growth rate decreased from 77.2% at baseline to 29.4%. There was no VS RR and 3 of 8 (37.5%) participants had stable disease. Decreased or unchanged VS volume after 3 months of treatment was predictive of stabilization at 12 months. Seven of eight participants had stable hearing during treatment except one with a decline in word recognition score. Ten of twelve participants reported only minimal changes to their QOL scores.

**Conclusions:**

Volumetric imaging at 3 months can serve as an early biomarker to predict long-term sensitivity to everolimus treatment. Everolimus may represent a safe treatment option to decrease the growth of NF2-related VS in patients who have stable hearing and neurological condition. TRN: NCT01345136 (April 29, 2011).

**Supplementary Information:**

The online version contains supplementary material available at 10.1007/s11060-024-04596-4.

## Introduction

*NF2*-related schwannomatosis (NF2) (formerly Neurofibromatosis type 2) is a rare autosomal-dominant tumor predisposition syndrome, with an incidence of 1 case in 33,000 live births [1–3]. NF2 patients develop multiple tumors including bilateral vestibular schwannomas (VS), associated with possible bilateral hearing loss, tinnitus, and/or balance disturbances, as well as schwannomas on other cranial and spinal nerves, meningiomas and ependymomas. The tumor burden usually presents early in life, but clinical management can be variable between different patients because of disease heterogeneity in clinical manifestations such as hearing loss and VS growth [4]. Standard management of NF2 includes conservative observation and microsurgical resection of growing and/or symptomatic tumors. However, surgical resection of VS is associated with a significant rate of hearing loss and carries risks for facial function deficits. While radiotherapy is an alternative therapeutic option in sporadic VS, it is generally not recommended as an early treatment modality for NF2 patients because of a small possible risk of malignant transformation [5]. Targeted agents have been used for the medical management of NF2-related VS [6–8]. Bevacizumab treatment is associated with high rates of hearing (70%) and tumor (89%) stability [9–11]. However, these VS will almost invariably progress and long-term side effects such as hypertension and proteinuria have been reported [12, 13]. Other targeted therapies for VS treatments, such as the endothelial growth factor receptor (EGFR) family kinase inhibitors, lapatinib and erlotinib have been used with limited success [14, 15].

Merlin, the NF2 protein, has been identified as a novel negative regulator of mammalian target of rapamycin complex 1 (mTORC1), with functional loss of merlin yielding activation of mTORC1 signaling in NF2-related tumors [16, 17]. Our previous study in NF2 mouse schwannoma models showed that long-term inhibition of mTORC1 by rapamycin inhibited tumorigenesis without significant toxicity [18]. Concomitant with these preclinical studies, we administered everolimus to an index patient with growing VS over a prolonged period resulting in a clinically meaningful growth delay [18]. Meanwhile, the rapalog everolimus was approved by the FDA for subependymal giant cell astrocytomas associated with tuberous sclerosis, another tumor predisposition syndrome with multiple benign tumors requiring long-term tumor treatment [19]. Two phase II clinical trials have further evaluated the efficacy of everolimus for progressive VS but only found the best response to be tumor stability [20, 21]. Altogether, these data led us to conduct this dual institution open-label phase II clinical trial to evaluate radiographic, hearing and quality of life (QOL) outcomes following everolimus treatment for NF2 patients with progressive VS. The results of the study performed in France were previously published [20]. Here, we report the results of the US study. In addition to response, we also performed a post-hoc analysis to evaluate whether imaging can serve as an early signature of tumor stabilization.

## Materials and methods

### Patient selection

Patients age 16 or older who met diagnostic criteria for NF2 (NIH 1988) and had progressive VS not requiring surgical resection or radiation therapy were eligible for the trial. We defined progressive VS as ≥ 20% volumetric increase on MRI in the prior 12 months. We excluded patients with prior radiation therapy within the past 60 months to eliminate delayed radiation treatment effects, as well as patients currently undergoing or having received anti-tumor therapy in the past 4 weeks. The full list of eligibility criteria is in Supplementary Information. All participants gave written informed consent before enrollment. Monitoring was assured by the Data Safety Monitoring Board DSMB of the UCLA Jonsson Comprehensive Cancer Center. Data, safety monitoring and results were subject to review by the FDA, UCLA IRB, and Novartis who provided the study drug. We received FDA approval for our protocol using RAD001 (everolimus) as an Investigational New Drug (IND #111195) in NF2.

### Drug administration and safety

Adult participants (18 and older) were started on everolimus at 10 mg/day while adolescents aged 16–17 were started on 3 mg/day. All participants could receive study drug once daily continuously for 52 weeks or until unacceptable toxicity as per investigator’s judgment or patient’s preference. Adverse events were delineated through the National Cancer Institute Common Toxicity Criteria (CTCAE 3.0) [22]. For adult participants who experienced grade 3 or higher or intolerable toxicity, everolimus could be reduced to 5 mg/day. To assess compliance and trend plasma levels, we performed pharmacokinetic drug studies at the University of California, Los Angeles.

### Radiologic measurement of tumor volume

Imaging was performed using a 3T clinical MRI scanner (Philips Healthcare, Los Angeles California). Tumor volume was determined using three-dimensional gradient echo T1-weighted post-gadolinium sequences with 1-mm slice thickness, no gap, by manual segmentation in OsiriX 4.1.2 software (OsiriX, Geneva, Switzerland) and MEDx 3.44 software (Medical Numerics, Inc., Germantown, MD), and independently measured by three investigators (ED, AS and MG). If volumes deviated by more than 5%, investigators jointly reviewed images for agreement. Pre-study enrollment tumor growth was determined using MRI within 12 months of baseline and a baseline MRI obtained within 28 days of treatment start. MRI was then performed every three months during treatment and three months after last dose. Study primary endpoint was change in the target VS volume by MRI from baseline to 12 months. RR was defined as tumor volume reduction of ≥ 20% from baseline, while progression was defined as tumor volume increase of ≥ 20% from baseline. Tumors that had < 20% volumetric increase or reduction were considered stable [23]. Time to tumor progression (TTP) was defined as the interpolated time needed for the tumor to increase 20% in volume before and during treatment [23]. To allow for a direct comparison of tumor growth rate among patients with pre-enrollment MRIs acquired at different time intervals, we calculated the annual growth rate (AGR) [11]. For consistency this measure was also applied to the different time points during the trial period. The annual growth rate (AGR) was calculated as the percentage change in tumor volume normalized to one year (%/year), using the formula: AGR = [((Vol_2_-Vol_1_)/Vol_1_) x 100]/(Date_2_-Date_1_) x 365, where Vol_1_ and Vol_2_ are the tumor volumes measured at current (Vol_1_) and previous (Vol_2_) MRI at dates Date_1_ and Date_2_, respectively.

### Hearing evaluation

Hearing outcomes were assessed using pure tone averages (PTA) and word recognition scores (WRS). PTA was defined as the average of individual threshold frequencies at 500, 1000, 2000 and 4000 Hz for each ear. Hearing deterioration was defined as an increase of more than 10 dB in PTA from baseline to the 12-month evaluation, while hearing improvement was categorized as a decrease of more than 10 dB in PTA. Per prior studies, WRS was evaluated using a 50 item recorded Central Institute for the Deaf CID-W22 monosyllable word list [21, 24, 25]. In addition to PTA, we also considered hearing deterioration as a decrease in WRS ≥ 20% from baseline to last everolimus treatment, while hearing improvement was defined as an increase in WRS ≥ 20%.

### Quality of life

We assessed QOL using three questionnaires [26–30] at baseline, and at 6-month and 12-month follow-ups. The SF-36 reports different domains of Health Related Qualify of Life (HRQOL) in the previous month, including physical limitations and general health perceptions [29]. The NF2 impact on QOL (NFTI-QOL) was specifically designed for NF2 patients [27, 31]. The tinnitus handicap inventory (THI) reports on tinnitus with three subscales on functional, emotional, and catastrophic responses [30, 32, 33].

### Statistical analysis

Baseline patient demographics and characteristics were summarized using descriptive statistics. TTP was calculated before treatment (reference to baseline time point) and during treatment (from initial date of everolimus to date of volumetric tumor progression) and represented using the Kaplan-Meier method. A Mann-Whitney test was used to compare AGR.

## Results

### Patient demographics, target tumor characteristics and response to treatment

**Fig. 1:**
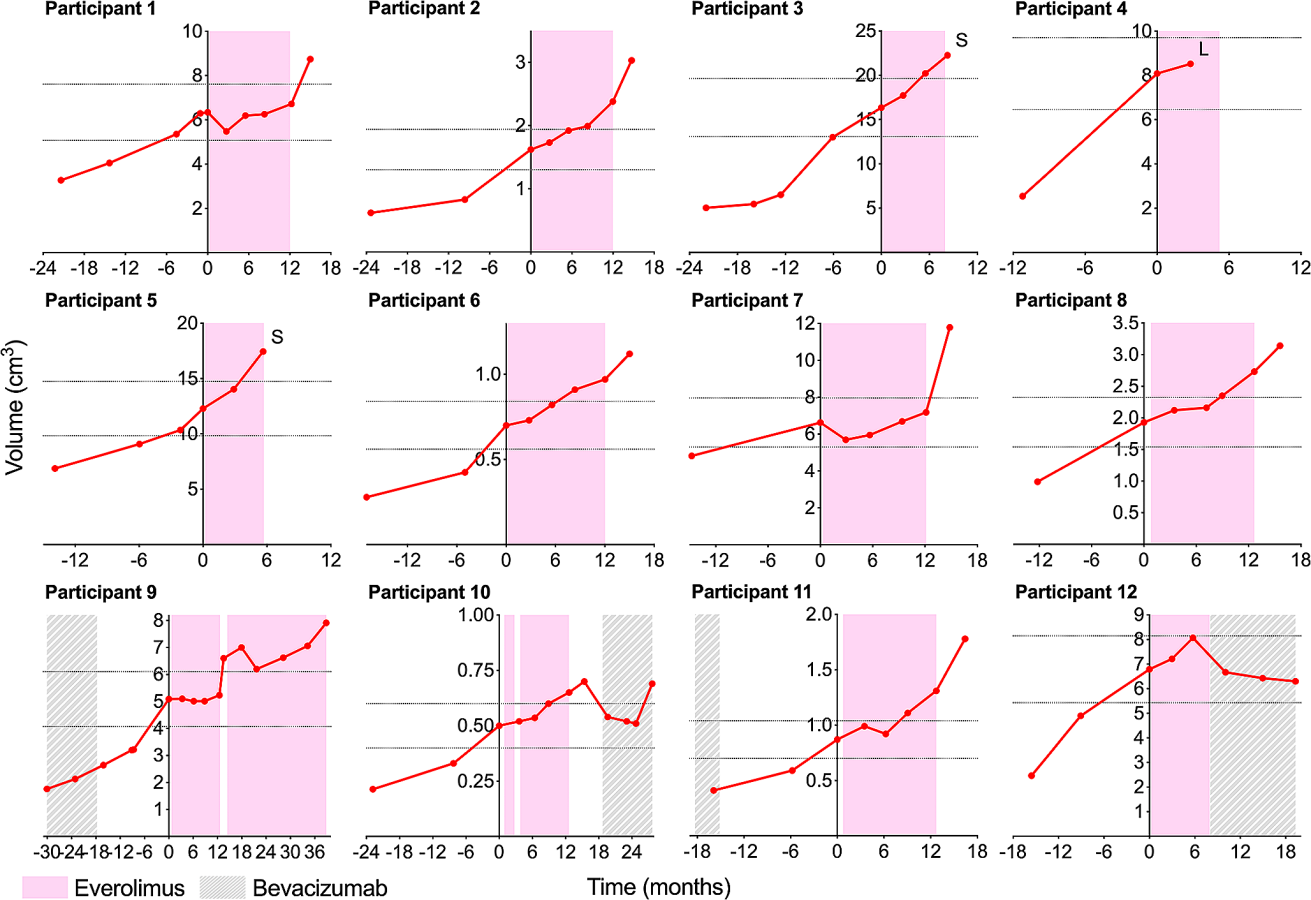
Volumetric change of target VS for the 12 participants before, during and after the clinical trial. Background color codes represent treatment periods for everolimus (*pink*) and bevacizumab (*gray*). The *horizontal dotted lines* represent +/-20% of each individual tumor volume compared to baseline tumor volume at the start of the trial. Four participants (3, 4, 5, 12) were taken off study. Two had surgical removal of target VS (*S*), one was lost to follow-up (*L*) and bevacizumab was started in another one

A total of twelve participants (9 F/3 M) were enrolled with an average age at start of study of 25.8 years (range: 16.3–45.6), including three < 18. Eight participants (1, 2, 6, 7, 8, 9, 10 and 11) completed the 12-month study treatment period (Table 1). Four participants (3, 4, 5, 12) were taken off study early because of significant tumor progression during treatment based on investigator’s evaluation, or patient’s preference (Fig. 1). At baseline, the median volume of the target VS was 5.7 cm^3^ (IQR 1.1 to 7.8). The median annual growth rate between the pre-baseline and baseline MRI was 77.2% (IQR 50.4 to 113.1), measured at a median time interval of 8.5 months (IQR 5.8 to 10.8). After 12 months of treatment, the median volume was 2.6 cm^3^ (IQR 1.1 to 6.4) and the median annual growth rate decreased to 29.4%/year (IQR 21.8 to 57.2) (Table 2). Overall, the best tumor response seen in all participants was -14% during the first 3 months of treatment for participant 7. Thus, the study did not reach the RR primary endpoint.

**Table 1:** Demographics and clinical characteristics of the 12 patients participating in the everolimus phase II trial

Participant No.	Institution	Sex	Age at drug treatment (years)	Final everolimus dosage (mg)	Other intracranial lesions	Spinal tumors	Completed trial	Stable^a^ tumor volume	Bevacizumab
1	HRI	F	25.7	10	Yes	No	Yes	Yes	-
2	HRI	F	21.8	7.5	Yes	No	Yes	No	-
3	HRI	F	30.9	10	Yes	No	No	No	-
4	HRI	F	18.0	10	Yes	Yes	No	No	-
5	HRI	F	20.2	15	Yes	No	No	No	-
6	HRI	F	16.3	10	Yes	No	Yes	No	-
7	HRI	F	45.6	10	Yes	No	Yes	Yes	-
8	HRI	M	16.8	10	Yes	No	Yes	No	-
9	UCLA	F	30.6	10	No	No	Yes	Yes	Pre-trial
10	UCLA	M	43.1	10	No	No	Yes	No	Post-trial
11	UCLA	F	18.1	12.5	Yes	No	Yes	No	Pre-trial
12	UCLA	M	22.3	10	Yes	Yes	No	No	Post-trial
Total or mean (% or range)		9 F (75%) 3 M (25%)	25.8 (16.3–45.6)	10.4 (7.5–12.5)	10 (83%)	2 (17%)	8 (67%)	3 (25%)	

HRI House Research Institute; UCLA University of California, Los Angeles; ^a^defined as tumors with less than a 20% volumetric increase or reduction

**Table 2:** Target tumor volumetric analysis and annual growth rates

Participant No.	Tumor target side	Volume at baseline (cm^3^)	AGR at baseline (%/year)	AGR at 3 months (%/year)	AGR at 6 months (%/year)	AGR at 9 months (%/year)	AGR at 12 months (%/year)	AGR at 15 months (%/year)
1	R	6.34	48.01	-58.94	56.30	4.16	22.87	130.62
2	R	1.62	118.17	29.86	47.16	15.66	63.87	118.67
3	R	16.34	50.23	37.14	60.34	44.08		
4	L	8.08	232.78	23.38				
5	R	12.28	71.86	57.49	105.54			
6	L	0.7	155.48	18.40	52.94	47.13	21.49	62.03
7	L	6.63	30.46	-58.15	19.86	40.17	31.83	281.74
8	L	1.93	93.16	33.90	6.09	58.38	52.70	60.91
9	R	5.09	79.69	0.71	-7.40	0.00	14.31	298.79
10	L	0.5	74.62	13.15	8.26	63.43	26.92	33.42
11	L	0.87	97.86	47.50	-30.72	87.65	58.72	115.89
12	L	6.79	50.83	24.81	51.83			
Median (IQR)		5.7 (1.1–7.8)	77.2 (50.4–113.1)	24.1 (3.8–36.3)	47.2 (6.11–56.3)	44.1 (9.9–60.9)	29.4 (21.8–57.2)	117.3 (61.2–244)

AGR Annual Growth Rate compared to previous scan; IQR Interquartile Range

**Fig. 2:**
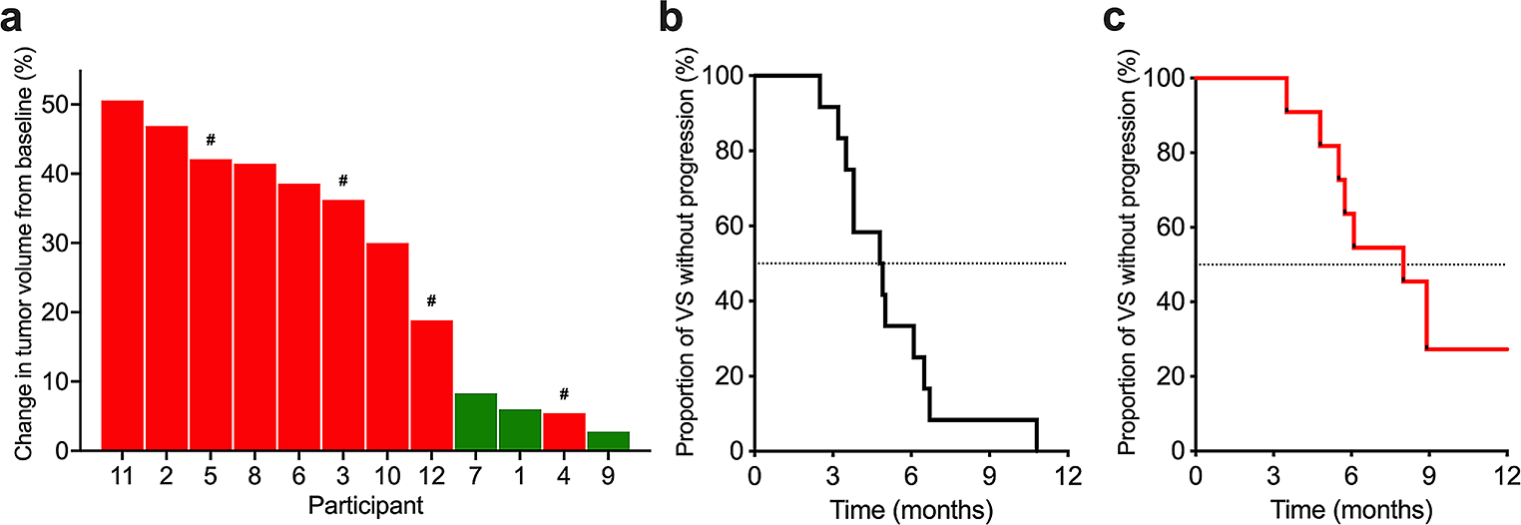
Volumetric change and time to progression of target VS for the 12 participants. **(a)** Change in target VS volume between the baseline and the end of the 12-month everolimus treatment or time when participant removed (#) from trial because of tumor progression (3, 5, 12) or voluntary withdrawal (4). *Green bars* show patients with stable disease and *red bars* patients with progressive disease. Time to progression of target VS before **(b)** and during **(c)** everolimus treatment. The median interpolated time to progression was 4.9 months before treatment and 8 months during everolimus treatment

During the 52-week treatment, 3 participants (1, 7 and 9; 25%) had stable disease with an average target VS volume increase of 5.7% at 12 months, compared to 38% increase at baseline (Fig. 2a). Participant 1 experienced rapid decreased hearing in the contralateral ear and balance deterioration one month after everolimus discontinuation, associated with marked growth of the target VS (30% in 3 months, 8.7 cm^3^) and non-target VS (23% in 3 months, 13.5 cm^3^) (Supp. Figure 1). The non-target VS was subsequently surgically removed. Participant 7 whose target VS was stable under treatment, showed a markedly rapid increase in tumor volume of 64%, 3 months after treatment discontinuation. The VS was subsequently surgically removed. Target VS of participant 9 was stable during treatment: MRIs at 3, 6, 9 and 12 months showed no increase in VS volume compared to the baseline. About two weeks after stopping the drug treatment, the patient noticed increased symptoms with hearing drum-like sounds without other complaints (all mouth ulcers had resolved within a few days after stopping the drug). After MRI confirmed tumor progression (26% in 1 month), the patient was rechallenged with everolimus treatment on a compassionate-use basis. After resuming everolimus treatment, the target VS showed growth stabilization (+ 6% compared to previous MRI) and WRS improved from 96 to 100 at month 15. Eventually, the tumor progressed (+ 20% compared to volume at rechallenge) 26 months after restarting drug treatment. Two patients (10 and 12) who had continuous tumor growth on everolimus subsequently received bevacizumab alone with some tumor shrinkage and overall tumor stability.

**Fig. 3:**
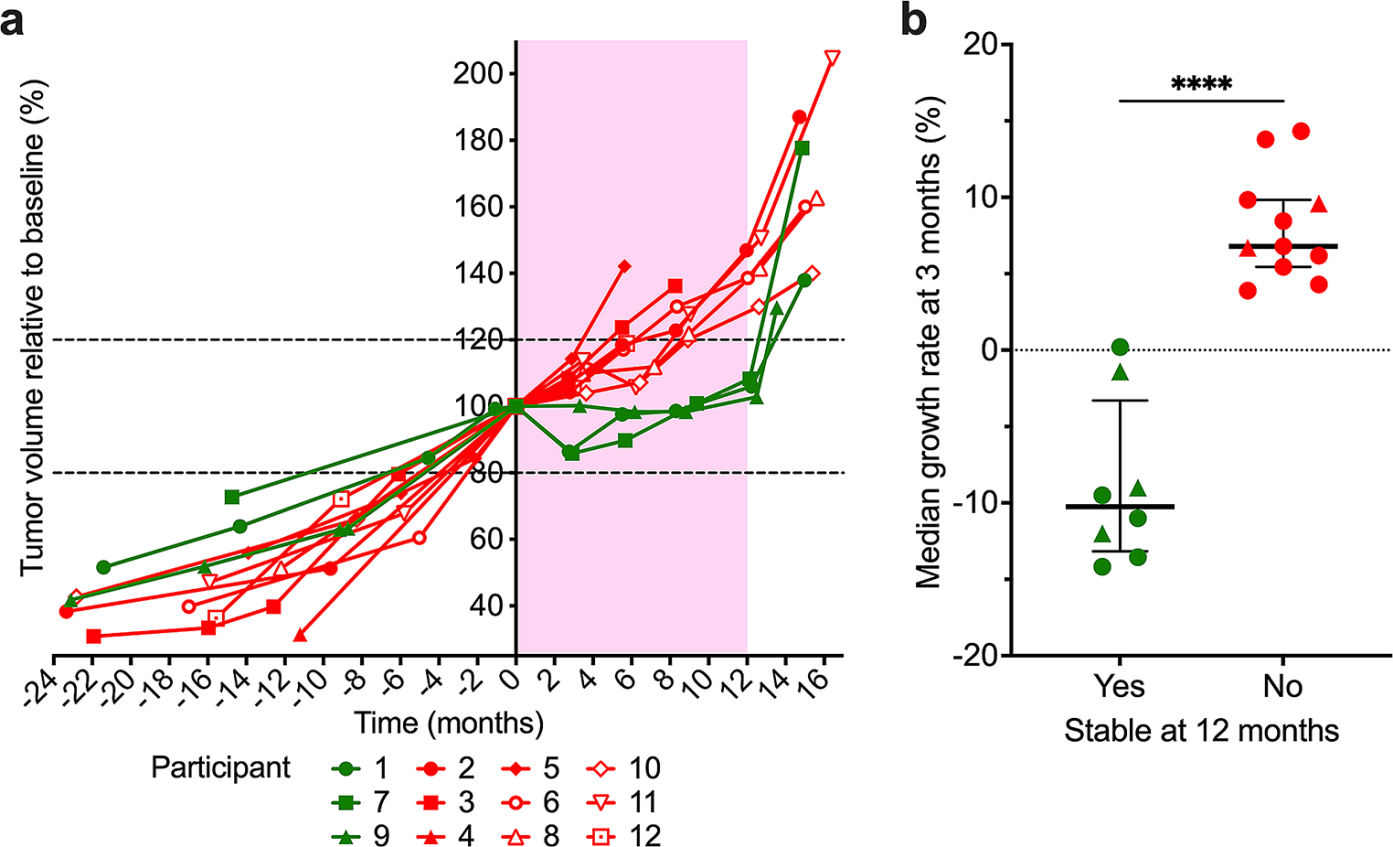
Change in tumor size over time and early prediction of sensitivity to everolimus. **(a)** Measurement of target VS volumes relative to baseline before, during (*pink background*) and after everolimus treatment showing participants with stable (*green lines*) or progressive (*red lines*) disease. The *horizontal dotted lines* represent +/-20% of tumor volume compared to baseline tumor volume. **(b)** Median of tumor growth rate after 3 months of continuous everolimus treatment statistically predicts the overall tumor volume response after 12 months of treatment in both UCLA (*circles*) and Pitié-Salpêtrière Hospital (*triangles*) participants. Error bars represent the interquartile range

An unplanned secondary analysis showed that the interpolated TTP for target VS was 4.9 months before treatment and improved to 8 months during study treatment (Fig. 2b, c), indicating that the TTP evolution slowed during treatment. Remarkably, early reduction of VS volume by everolimus was predictive of tumor stabilization at drug trial completion. Three participants (1, 7, 9) with no tumor growth and/or tumor reduction at 3 months (Fig. 3a, b) demonstrated stable disease at 12 months. In contrast, patients who exhibited any increase in tumor volume, even insignificant growth (< 20%), at 3 months showed tumor progression (> 20%) at the 12-month MRI follow-up (Fig. 3a). Retrospective analysis of tumor growth data from a French study of everolimus [20] also corroborated this observation, showing that participants with stable VS at the 12 month MRI showed a volume reduction or no growth at the 3-month MRI (Fig. 3b). Thus, the absence of tumor growth at 3 months of continuous everolimus treatment was predictive of stable response at 12 months.

### Non-target tumors

In addition to target VS, we analyzed volumes for six contralateral VS, eight intra-cranial meningiomas, one C1 root schwannoma and one ependymoma. We did not observe any imaging responses, and 11 of 15 these non-target tumors were stable during the study period (Supp. Figure 1). However, participant 6 showed a 6.7% volumetric reduction in the ependymoma, and participant 8 a 6.2% reduction in the contralateral non-target VS, with concurrent hearing recovery in the associated ear. A non-target meningioma in participant 12 was reduced by 9.6% in volume at 6 months compared to baseline.

### Hearing response

PTA remained stable or improved in all target ears of the 8 participants who completed the 12-months study treatment (1, 2, 6, 7, 8, 9, 10, 11) and WRS remained stable or improved for 7 of these 8 participants (Supp. Figure 2). Despite target VS stabilization, participant 7 experienced marked hearing deterioration from 58 to 20% WRS over the drug treatment period, decreasing to 0% after drug withdrawal at the 15-month time point. Participant 8 showed stabilization of the non-target VS of -6% at 12 months, which coincided with improvement in hearing outcomes from PTA of 60dB at baseline to 52.5dB at 12-month follow-up.

### Drug toxicity

All enrolled patients underwent toxicity and safety monitoring. No participants withdrew from the study due to drug toxicity (Supp. Table 1). However, participant 4 had a grade 3 related adverse event of pneumonia, which required hospitalization and antibiotic therapy. Participant 10 experienced a severe grade 4 elevated triglycerides and consequently treated with statin. One patient had a dose reduction for intolerable grade 2 oral ulcers. The most common side effects included: mouth ulcers (*n* = 9), fatigue (*n* = 6), acne (*n* = 6), skin rashes (*n* = 5), and headache (*n* = 5).

### Quality of life

All 12 participants completed the HRQOL questionnaire at baseline with 10 and 8 responses at six and twelve months, respectively. At baseline before the trial, patients with NF2 tended to have lower baseline QOL than literature normal values, which can be attributed to the effect of tumor burden interfering with daily functional activities [26–28]. The SF-36 questionnaire revealed that participants endorsed difficulty with fatigue (score = 58.3) and general health (69.2) (Supp. Figure 3a). In the NF2-specific QOL questionnaire (NFTI-QOL) participants most frequently reported problems with dizziness (score = 1.41), hearing (1.41), and vision (1.17) at initial evaluation (Supp. Figure 3b). The THI questionnaire showed that 10 of 12 participants (91.7%) experienced “no or very mild handicap” from their tinnitus (Supp. Figure 3c). While on clinical trial with everolimus, participants reported minimal worsening of SF-36 QOL over 12 months in most domains, except in emotional role (mean change = -19.4), fatigue (-10.2), social function (-11.5), pain (-11.5) and general health (-19.2). After 52 weeks of treatment, participants had negligible changes in their QOL scores, except for 2 participants. Participants 1 and 8 showed significant changes in their domain score for the SF36 questionnaire, but it is unclear if tumor progression was responsible for QOL decline, rather than the trial drug itself. Similarly, the NFTI-QOL questionnaire also demonstrated minimal impairment during the trial. The THI questionnaire detected higher grade tinnitus in a maximum of only 3 participants over the 12 months of treatment, likely responsible for their overall lower QOL scores, including in the THI subscales (Supp. Figure 3d) and NFTI-QOL hearing domain (Supp. Figure 3b).

## Discussion

Most current studies for progressive NF2-associated VS define success of a treatment based on tumor shrinkage. However, in a syndrome where tumors are slow growing and neurological and hearing deficits can take years to manifest, treatment with a low toxicity agent, which can inhibit tumor growth and induce prolonged tumor stabilization, may be as desirable as agents promoting tumor shrinkage. Concordant with prior trials, our results corroborate the cytostatic effect of everolimus on tumor growth [21, 34]. Because of its convenient oral administration and safety profile, everolimus should be considered for treatment-naïve patients or perhaps for those patients with difficulty traveling to a certified NF2 center offering clinical trial options. Practically, everolimus can serve as another therapeutic modality to delay time to bevacizumab or surgical intervention, temporarily avoiding their associated morbidities.

Vestibular schwannoma growth patterns can vary during progression [35, 36]. Currently, the recommended endpoint for NF2 clinical trials is to assess the percentage change in tumor growth over time based on patients’ natural history of tumor growth before starting the trial [8]. Our study shows that seven patients had reduced VS growth rate after 12 months of treatment. Overall, the TTP also improved with everolimus treatment by 3.1 months, comparing TTP before and during treatment. We found that an unchanged or decreased VS volume with an annualized VS growth rate of < 1% at 3 months into treatment was predictive of VS stabilization at 12 months. That is, imaging at 3 months allowed identification of patients displaying stable tumor growth along the entire treatment course up to 12 months. Moreover, in non-responders the risk of rebound growth following treatment cessation is less severe [34]. Thus, tumor response or tumor growth rate at 3 months can then serve as an early prognostic marker for prolonged sensitivity to everolimus, eventually allowing for rapid transition to an alternative therapeutic strategy.

Though it is unclear why everolimus response is variable, this may be attributed to the VS genomic and cellular heterogeneity potentially affecting drug distribution and target modulation [37–41]. Recent observations highlighted the potential for intrinsic schwannoma heterogeneity to influence the microenvironment and consequent multifaceted impact of everolimus [42]. We found that everolimus may also stabilize non-target tumors in NF2 patients. Overall, 11 of 15 non-target tumors were stable during treatment, likely due to their low or non-proliferative status. These non-target tumor results are corroborated by Karajannis et al. in their initial phase II trial of everolimus for NF2, noting a volume decrease in a cervical nerve root tumor [21].

Treatment with everolimus did not significantly alter QOL and hearing remained stable during treatment, except one participant with a decline in word recognition score. The therapy is tolerable in our patient population with minimal side effects. In general, our patients experienced the common and manageable side effects of stomatitis and fatigue, but we did not see severe adverse events such as non-infectious pneumonitis, wound healing issues, renal failure, or metabolic issues as reported in other studies of everolimus [43].

In summary, everolimus should be considered as an early treatment for NF2 patients with VS progression in the absence of significant symptoms or deficits requiring surgical or medical interventions (i.e. bevacizumab). Longer follow-up time and larger patient cohorts will be necessary for studies aimed to explore the effect of drug treatments on tumor growth and hearing trajectories.

## Conclusions

Although everolimus did not lead to a sustained RR in progressive NF2-associated VS in our study, we confirmed its growth rate inhibitory effects in a subset of patients. This effect can be predicted by MR imaging and volumetric analysis just after the first 3 months of treatment. As a result, everolimus may best serve as a first-line agent for treatment of naïve NF2 patients presenting with progressive VS but otherwise stable clinical symptoms due to its oral advantage and tolerable adverse event profile.

### Electronic supplementary material

Below is the link to the electronic supplementary material.

Supplementary Material 1

## Data Availability

Data generated and/or analyzed during the current study are available from the corresponding author on reasonable request.

## References

[1] Evans DR (2009). Neurofibromatosis type 2 (NF2): a clinical and molecular review. Orphanet J Rare Dis.

[2] Moualed D, Wong J, Thomas O, Heal C, Saqib R, Choi C, Lloyd S, Rutherford S, Stapleton E, Hammerbeck-Ward C, Pathmanaban O, Laitt R, Smith M, Wallace A, Kellett M, Evans G, King A, Freeman S (2022). Prevalence and natural history of schwannomas in neurofibromatosis type 2 (NF2): the influence of pathogenic variants. Eur J Hum Genet.

[3] Plotkin SR, Messiaen L, Legius E, Pancza P, Avery RA, Blakeley JO, Babovic-Vuksanovic D, Ferner R, Fisher MJ, Friedman JM, Giovannini M, Gutmann DH, Hanemann CO, Kalamarides M, Kehrer-Sawatzki H, Korf BR, Mautner VF, MacCollin M, Papi L, Rauen KA, Riccardi V, Schorry E, Smith MJ, Stemmer-Rachamimov A, Stevenson DA, Ullrich NJ, Viskochil D, Wimmer K, Yohay K, International Consensus Group on Neurofibromatosis Diagnostic, Huson C, Wolkenstein SM, Evans P (2022) DG Updated diagnostic criteria and nomenclature for neurofibromatosis type 2 and schwannomatosis: an international consensus recommendation. Genet Med 24:1967–1977 10.1016/j.gim.2022.05.007 10.1016/j.gim.2022.05.007 35674741

[4] Peyre M, Goutagny S, Bah A, Bernardeschi D, Larroque B, Sterkers O, Kalamarides M (2013). Conservative management of bilateral vestibular schwannomas in neurofibromatosis type 2 patients: hearing and tumor growth results. Neurosurgery.

[5] Evans DG, Halliday D, Obholzer R, Afridi S, Forde C, Rutherford SA, Hammerbeck-Ward C, Lloyd SK, Freeman SM, Pathmanaban ON, Thomas OM, Laitt RD, Stivaros S, Kilday JP, Vassallo G, McBain C, Lavin T, Paterson C, Whitfield G, McCabe MG, Axon PR, Halliday J, Mackeith S, Parry A, English Specialist NFRG, Harkness EF, Buttimore J, King AT (2023). Radiation treatment of benign tumors in NF2-related-schwannomatosis: a national study of 266 irradiated patients showing a significant increase in malignancy/malignant progression. Neurooncol Adv.

[6] Cumpston EC, Rhodes SD, Yates CW (2023). Advances in targeted therapy for neurofibromatosis type 2 (NF2)-Associated vestibular schwannomas. Curr Oncol Rep.

[7] Donna M, Phioanh Leia N, Francesco S, Raffaella M (2019). Neurofibromatosis type 2: current trends and future directions for targeted Biologic therapies. Neurofibromatosis.

[8] Evans DG, Kalamarides M, Hunter-Schaedle K, Blakeley J, Allen J, Babovic-Vuskanovic D, Belzberg A, Bollag G, Chen R, DiTomaso E, Golfinos J, Harris G, Jacob A, Kalpana G, Karajannis M, Korf B, Kurzrock R, Law M, McClatchey A, Packer R, Roehm P, Rubenstein A, Slattery W, Tonsgard JH, Welling DB, Widemann B, Yohay K, Giovannini M (2009). Consensus recommendations to accelerate clinical trials for neurofibromatosis type 2. Clin Cancer Res.

[9] Plotkin SR, Allen J, Dhall G, Campian JL, Clapp DW, Fisher MJ, Jain RK, Tonsgard J, Ullrich NJ, Thomas C, Edwards LJ, Korf B, Packer R, Karajannis MA, Blakeley JO (2023). Multicenter, prospective, phase II study of maintenance bevacizumab for children and adults with NF2-related schwannomatosis and progressive vestibular schwannoma. Neuro Oncol.

[10] Plotkin SR, Duda DG, Muzikansky A, Allen J, Blakeley J, Rosser T, Campian JL, Clapp DW, Fisher MJ, Tonsgard J, Ullrich N, Thomas C, Cutter G, Korf B, Packer R, Karajannis MA (2019). Multicenter, prospective, phase II and Biomarker Study of High-Dose Bevacizumab as induction therapy in patients with neurofibromatosis type 2 and progressive vestibular Schwannoma. J Clin Oncol.

[11] Plotkin SR, Stemmer-Rachamimov AO, Barker FG, Halpin C, Padera TP, Tyrrell A, Sorensen AG, Jain RK, di Tomaso E (2009). Hearing improvement after bevacizumab in patients with neurofibromatosis type 2. N Engl J Med.

[12] Killeen DE, Klesse L, Tolisano AM, Hunter JB, Kutz JW (2019). Long-Term effects of Bevacizumab on vestibular Schwannoma volume in neurofibromatosis type 2 patients. J Neurol Surg B.

[13] Slusarz KM, Merker VL, Muzikansky A, Francis SA, Plotkin SR (2014). Long-term toxicity of bevacizumab therapy in neurofibromatosis 2 patients. Cancer Chemother Pharmacol.

[14] Karajannis MA, Legault G, Hagiwara M, Ballas MS, Brown K, Nusbaum AO, Hochman T, Goldberg JD, Koch KM, Golfinos JG, Roland JT, Allen JC (2012). Phase II trial of lapatinib in adult and pediatric patients with neurofibromatosis type 2 and progressive vestibular schwannomas. Neuro Oncol.

[15] Plotkin SR, Halpin C, McKenna MJ, Loeffler JS, Batchelor TT, Barker FG (2010). Erlotinib for progressive vestibular schwannoma in neurofibromatosis 2 patients. Otol Neurotol.

[16] James MF, Han S, Polizzano C, Plotkin SR, Manning BD, Stemmer-Rachamimov AO, Gusella JF, Ramesh V (2009). NF2/merlin is a novel negative regulator of mTOR complex 1, and activation of mTORC1 is associated with meningioma and schwannoma growth. Mol Cell Biol.

[17] James MF, Stivison E, Beauchamp R, Han S, Li H, Wallace MR, Gusella JF, Stemmer-Rachamimov AO, Ramesh V (2012). Regulation of mTOR complex 2 signaling in neurofibromatosis 2-deficient target cell types. Mol cancer Research: MCR.

[18] Giovannini M, Bonne NX, Vitte J, Chareyre F, Tanaka K, Adams R, Fisher LM, Valeyrie-Allanore L, Wolkenstein P, Goutagny S, Kalamarides M (2014). mTORC1 inhibition delays growth of neurofibromatosis type 2 schwannoma. Neuro Oncol.

[19] Franz DN, Belousova E, Sparagana S, Bebin EM, Frost M, Kuperman R, Witt O, Kohrman MH, Flamini JR, Wu JY, Curatolo P, de Vries PJ, Whittemore VH, Thiele EA, Ford JP, Shah G, Cauwel H, Lebwohl D, Sahmoud T, Jozwiak S (2013). Efficacy and safety of everolimus for subependymal giant cell astrocytomas associated with tuberous sclerosis complex (EXIST-1): a multicentre, randomised, placebo-controlled phase 3 trial. Lancet.

[20] Goutagny S, Raymond E, Esposito-Farese M, Trunet S, Mawrin C, Bernardeschi D, Larroque B, Sterkers O, Giovannini M, Kalamarides M (2015). Phase II study of mTORC1 inhibition by everolimus in neurofibromatosis type 2 patients with growing vestibular schwannomas. J Neurooncol.

[21] Karajannis MA, Legault G, Hagiwara M, Giancotti FG, Filatov A, Derman A, Hochman T, Goldberg JD, Vega E, Wisoff JH, Golfinos JG, Merkelson A, Roland JT, Allen JC (2014). Phase II study of everolimus in children and adults with neurofibromatosis type 2 and progressive vestibular schwannomas. Neuro Oncol.

[22] Trotti A, Colevas AD, Setser A, Rusch V, Jaques D, Budach V, Langer C, Murphy B, Cumberlin R, Coleman CN, Rubin P (2003). CTCAE v3.0: development of a comprehensive grading system for the adverse effects of cancer treatment. Semin Radiat Oncol.

[23] Dombi E, Ardern-Holmes SL, Babovic-Vuksanovic D, Barker FG, Connor S, Evans DG, Fisher MJ, Goutagny S, Harris GJ, Jaramillo D, Karajannis MA, Korf BR, Mautner V, Plotkin SR, Poussaint TY, Robertson K, Shih CS, Widemann BC, Collaboration REI (2013). Recommendations for imaging tumor response in neurofibromatosis clinical trials. Neurology.

[24] Plotkin SR, Halpin C, Blakeley JO, Slattery WH, Welling DB, Chang SM, Loeffler JS, Harris GJ, Sorensen AG, McKenna MJ, Barker FG (2009). Suggested response criteria for phase II antitumor drug studies for neurofibromatosis type 2 related vestibular schwannoma. J Neurooncol.

[25] Blakeley JO, Evans DG, Adler J, Brackmann D, Chen R, Ferner RE, Hanemann CO, Harris G, Huson SM, Jacob A, Kalamarides M, Karajannis MA, Korf BR, Mautner VF, McClatchey AI, Miao H, Plotkin SR, Slattery W, Stemmer-Rachamimov AO, Welling DB, Wen PY, Widemann B, Hunter-Schaedle K, Giovannini M (2012). Consensus recommendations for current treatments and accelerating clinical trials for patients with neurofibromatosis type 2. Am J Med Genet A.

[26] Merker VL, Bergner AL, Vranceanu AM, Muzikansky A, Slattery W, Plotkin SR (2016). Health-related quality of life of individuals with neurofibromatosis type 2: results from the NF2 Natural History Study. Otol Neurotol.

[27] Hornigold RE, Golding JF, Leschziner G, Obholzer R, Gleeson MJ, Thomas N, Walsh D, Saeed S, Ferner RE (2012). The NFTI-QOL: a Disease-Specific Quality of Life Questionnaire for neurofibromatosis 2. J Neurol Surg Part B Skull base.

[28] Ferner RE, Shaw A, Evans DG, McAleer D, Halliday D, Parry A, Raymond FL, Durie-Gair J, Hanemann CO, Hornigold R, Axon P, Golding JF (2014). Longitudinal evaluation of quality of life in 288 patients with neurofibromatosis 2. J Neurol.

[29] Ware JE, Sherbourne CD (1992). The MOS 36-item short-form health survey (SF-36). I. conceptual framework and item selection. Med Care.

[30] Newman CW, Jacobson GP, Spitzer JB (1996). Development of the Tinnitus Handicap Inventory. Archives otolaryngology–head neck Surg.

[31] Wolters PL, Vranceanu AM, Thompson HL, Martin S, Merker VL, Baldwin A, Barnett C, Koetsier KS, Hingtgen CM, Funes CJ, Tonsgard JH, Schorry EK, Allen T, Smith T, Franklin B, Reeve S, Collaboration REI (2021). Current recommendations for patient-reported outcome measures assessing domains of quality of life in neurofibromatosis clinical trials. Neurology.

[32] McCombe A, Baguley D, Coles R, McKenna L, McKinney C, Windle-Taylor P, British Association of, Otolaryngologists H, Neck S (2001) Guidelines for the grading of tinnitus severity: the results of a working group commissioned by the British Association of Otolaryngologists, Head and Neck Surgeons, 1999. Clin Otolaryngol Allied Sci 26:388–393 10.1046/j.1365-2273.2001.00490.x 10.1046/j.1365-2273.2001.00490.x 11678946

[33] Newman CW, Sandridge SA, Jacobson GP (1998). Psychometric adequacy of the Tinnitus Handicap Inventory (THI) for evaluating treatment outcome. J Am Acad Audiol.

[34] Goutagny S, Giovannini M, Kalamarides M (2017). A 4-year phase II study of everolimus in NF2 patients with growing vestibular schwannomas. J Neurooncol.

[35] Dirks MS, Butman JA, Kim HJ, Wu T, Morgan K, Tran AP, Lonser RR, Asthagiri AR (2012). Long-term natural history of neurofibromatosis type 2-associated intracranial tumors. J Neurosurg.

[36] Peyre M, Bernardeschi D, Sterkers O, Kalamarides M (2018). Natural history of vestibular schwannomas and hearing loss in NF2 patients. Neurochirurgie.

[37] Agnihotri S, Jalali S, Wilson MR, Danesh A, Li M, Klironomos G, Krieger JR, Mansouri A, Khan O, Mamatjan Y, Landon-Brace N, Tung T, Dowar M, Li T, Bruce JP, Burrell KE, Tonge PD, Alamsahebpour A, Krischek B, Agarwalla PK, Bi WL, Dunn IF, Beroukhim R, Fehlings MG, Bril V, Pagnotta SM, Iavarone A, Pugh TJ, Aldape KD, Zadeh G (2016). The genomic landscape of schwannoma. Nat Genet.

[38] Breshears JD, Liu JS, Vasudevan H, Pekmezci M, Castro MRH, Lang U, Chen W, Choudhury A, Magill ST, Braunstein S, Gopinath C, Nakamura JL, Sneed P, Perry A, McDermott MW, Villanueva-Meyer JE, Raleigh DR, Theodosopoulos PV (2019). Multiplatform Molecular profiling of vestibular Schwannoma reveals 2 subgroups of tumors with distinct Radiographic features and a methylation-based predictor of local recurrence. Neurosurgery.

[39] Karajannis MA, Mauguen A, Maloku E, Xu Q, Dunbar EM, Plotkin SR, Yaffee A, Wang S, Roland JT, Sen C, Placantonakis DG, Golfinos JG, Allen JC, Vitanza NA, Chiriboga LA, Schneider RJ, Deng J, Neubert TA, Goldberg JD, Zagzag D, Giancotti FG, Blakeley JO (2021). Phase 0 Clinical Trial of Everolimus in patients with vestibular Schwannoma or Meningioma. Mol Cancer Ther.

[40] Oya S, Yoshida S, Hanakita S, Inoue M (2022). Quantitative evaluation of proliferative potential using Flow Cytometry reveals Intratumoral Heterogeneity and its relevance to tumor characteristics in Vestibular Schwannomas. Curr Oncol.

[41] Roberts D, Maurya R, Takemon Y, Vitte J, Zhao J, Wong C-H, Slattery W, Peng K, Lekovic G, Schwartz M, Bulsara K, Ngan C, Giovannini M, Wei C-L (2019) Linked-read sequencing analysis reveals tumor-specific genome variation landscapes in neurofibromatosis type 2 (NF2) patients. Otology Neurotology: Official Publication Am Otological Soc Am Neurotology Soc [and] Eur Acad Otology Neurotology 40(e150–e159). 10.1097/MAO.0000000000002096 10.1097/MAO.0000000000002096 30624408

[42] Chiasson-MacKenzie C, Vitte J, Liu CH, Wright EA, Flynn EA, Stott SL, Giovannini M, McClatchey AI (2023). Cellular mechanisms of heterogeneity in NF2-mutant schwannoma. Nat Commun.

[43] Afinitor [package insert] (2022) Novartis Pharmaceuticals Corporation, East Hanover, New Jersey

