# Supplementary material for: Imaging as an early biomarker to predict sensitivity to everolimus for progressive NF2-related vestibular schwannoma
Source: J Neurooncol. 2024 Feb 19;167(2):339–48. doi: 10.1007/s11060-024-04596-4 (PMC11023969; doi:10.1007/s11060-024-04596-4)
Supplement: Supplementary file 1 — Supplementary Material 1 [file 11060_2024_4596_MOESM1_ESM.pdf]

Imaging as an early biomarker to predict sensitivity to everolimus for progressive NF2-related vestibular schwannoma

Phioanh L. Nghiemphu, Jeremie Vitte, Eva Dombi, Thien Nguyen, Naveed Wagle, Akira Ishiyama, Ali R. Sepahdari, David Cachia, Brigitte C. Widemann, Derald E. Brackmann, Joni K. Doherty, Michel Kalamarides, Marco Giovannini

Supplementary Information:

Eligibility Criteria:

Inclusion Criteria:
- Diagnosis of NF2 by National Institutes of Health (NIH) criteria
- Age ≥ 16 years
- Progressive VS growth during the previous 12 months.
- WHO performance status > or = 2
- Adequate bone marrow, liver and renal function.
- For women of childbearing potential, no pregnancy or breast-feeding
- Willingness and ability to comply with scheduled visits, drug administration plan, laboratory tests, other study procedures, and study restrictions.
- Willingness to provide informed consent

Exclusion Criteria:
- Inability to tolerate periodic MRI scans or gadolinium contrast.
- Inability to tolerate periodic audiologic testing or to understand a language with established scoring for word recognition testing.
- Inability to adequately perform volumetric measurement of at least 1 target lesionNote: Patients with cochlear or auditory brainstem implants may participate if a target lesion can be accurately assessed.
- Radiation therapy for the target lesion in the 60 months preceding inclusion in the study.
- Patients currently receiving anticancer therapies or who have received anticancer therapies within 4 weeks of the start of study drug.
- Immunization with attenuated live vaccines within one week of study entry or during study period.
- Presence of a fungal infection requiring systemic antifungal treatment at enrollment
- Other malignancies within the past 3 years except for adequately treated carcinoma of the cervix or basal or squamous cell carcinomas of the skin.
- Patients who have any severe and/or uncontrolled medical conditions.
- Patients with a known hypersensitivity to everolimus or other types of rapamycin or to its excipients.
- Patients unwilling to or unable to comply with the protocol

Supplementary Table 1. All reported toxic effects by CTCAE grade possibly or probably related to everolimus treatment

Adverse event	Grade 1 (n)	Grade 2 (n)	Grade 3 (n)	Grade 4 (n)	Total number of participants with adverse event (n)
Mouth Ulcers	7	2			9
Fatigue	6				6
Acne	6				6
Skin Rash	2	3			5
Abdominal Pain	2	1			3
Nausea	2	1			3
Insomnia	3				3
Dry Mouth	2				2
Hair Loss	2				2
Loss of appetite	1	1			2
Constipation	2				2
Vivid Dreams	2				2
Pneumonia			1		1
Elevated Triglycerides				1	1
Cold Sore		1			1
UTI		1			1
Pharyngitis		1			1
Candidiasis of mouth (Mucositis)		1			1
Sinus Headache	1				1
Confusion	1				1
Clumsiness	1				1
Emotional Blunting	1				1
Vertigo	1				1
Edema	1				1
Pain Throat	1				1
Fever	1				1
Lymph node pain	1				1
Chills	1				1

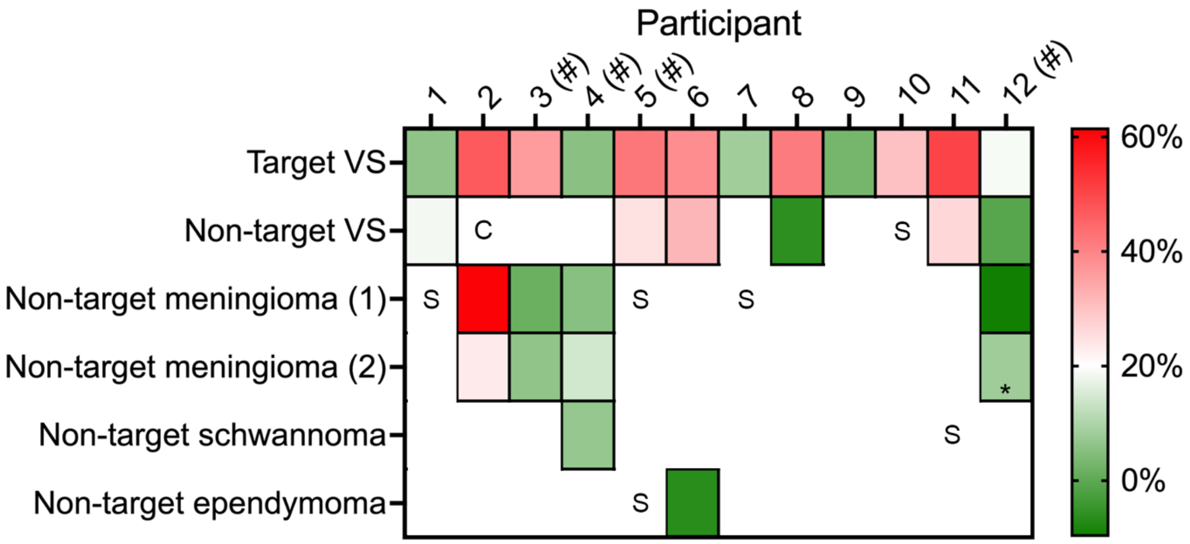

Supplementary Fig. 1 Change in volume of target VS and non-target tumors between the baseline and the end of the 12-month everolimus treatment or the last mesurement when drug was discontinued for participants 3, 4, 5 and 12 (#).*Participant 12 had one additional collision meningioma and two additional too-small-to-measure meningiomas; C: Collision tumor; S: tumor too small to measure

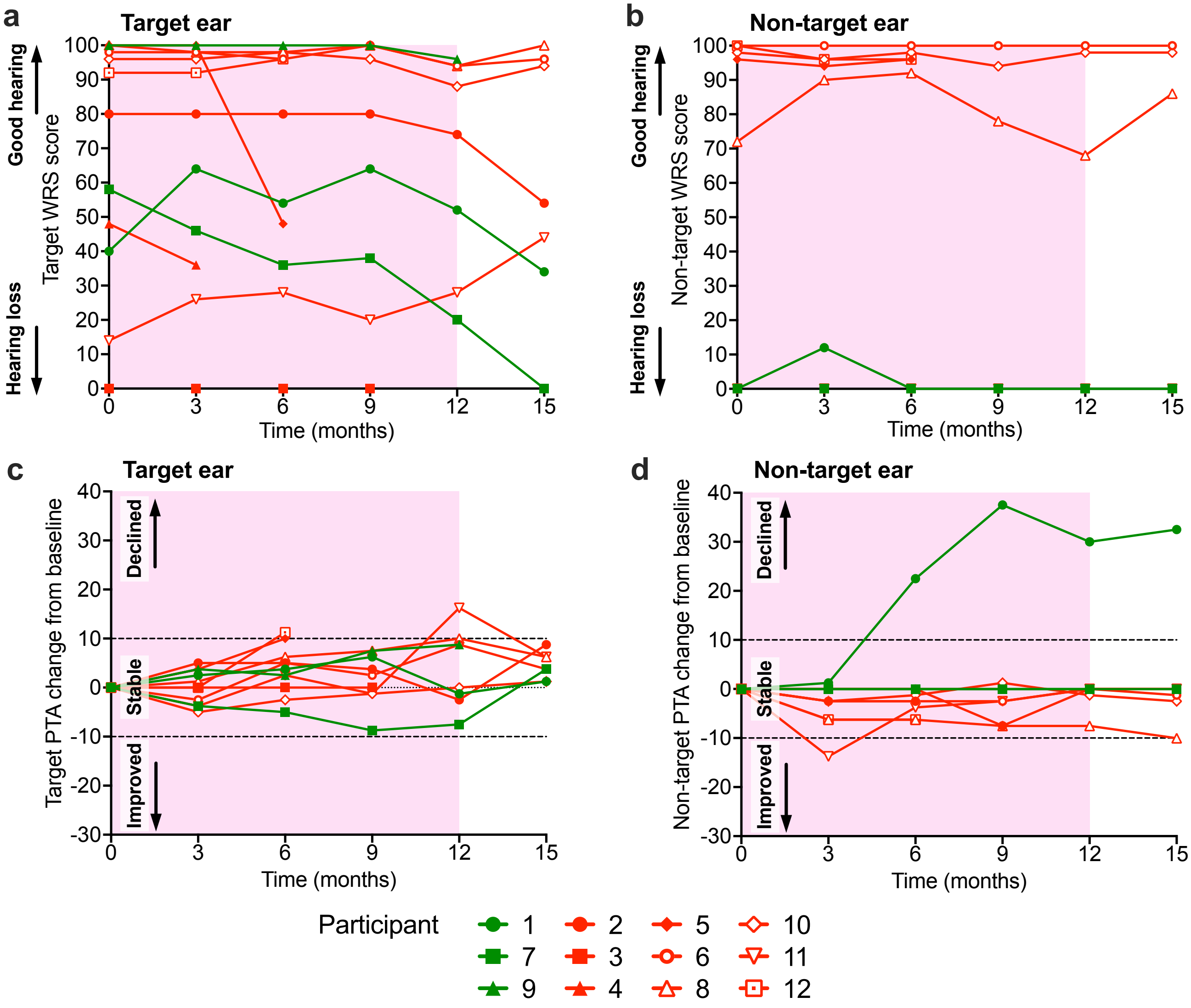

Supplementary Fig. 2 Assessment of speech intelligibility and hearing sensitivity of the target and non-target ears for the 12 participants. Measurement of speech intelligibility by Word Recognition Score (WRS) in target (**a**) and non-target (**b**) ears during everolimus treatment (*pink background*) and at the last visit of the clinical trial. Higher scores represent better hearing discrimination. Variations of the Pure Tone Average (PTA), measuring hearing sensitivity, in target (**c**) and non-target (**d**) ears between each visit and the baseline. PTA variation >10% represent a decrease in hearing sensitivity. *Green* and *red lines* represent participants with stable and progressive disease, respectively

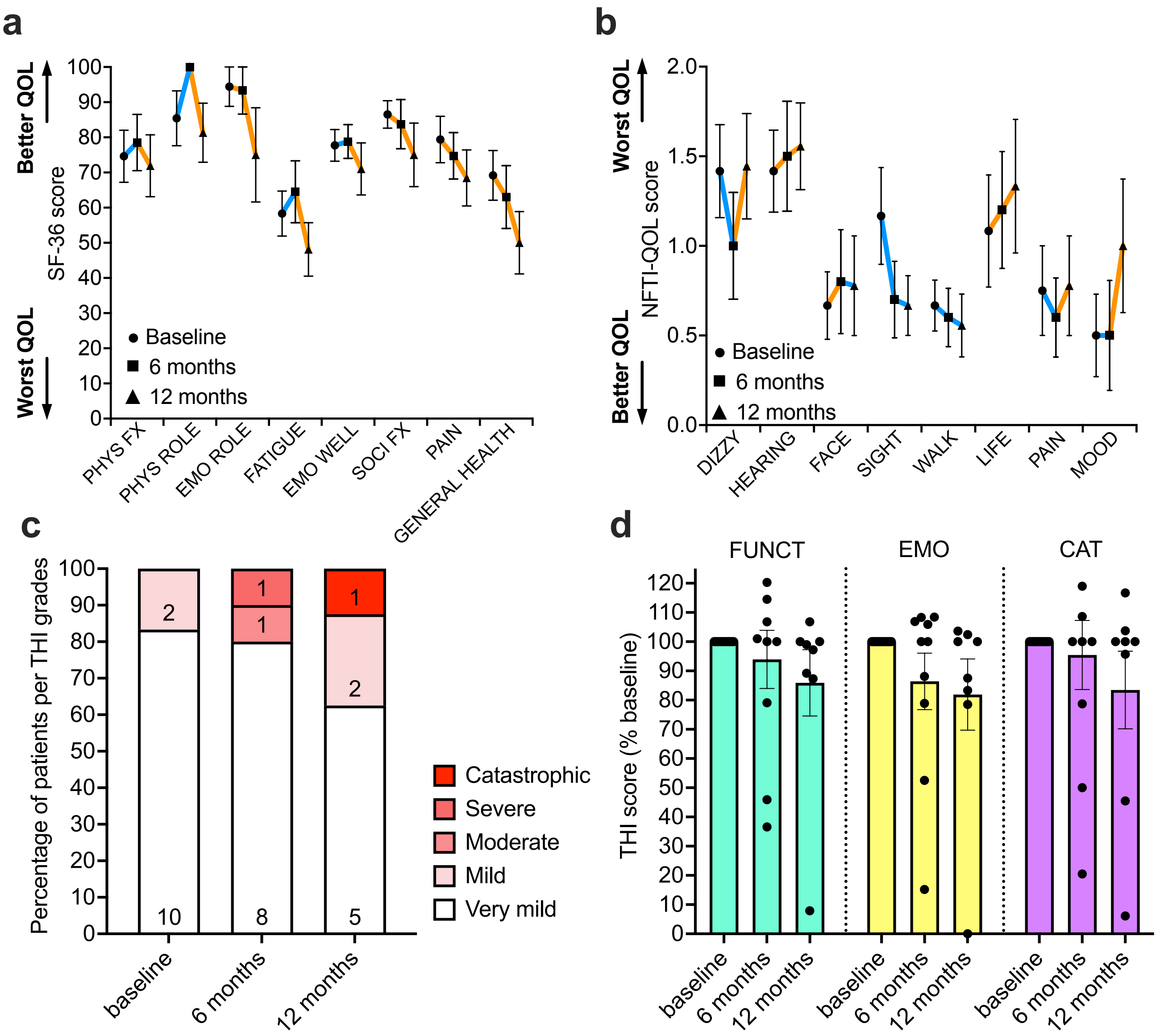

Supplementary Fig. 3 Changes in quality of life (QOL) for the 12 participants during the everolimus clinical trial. Changes in SF-36 scores (**a**) and NFTI-QOL scores (**b**) per domain at baseline, 6 and 12 months of treatment. Data from the SF-36 were normalized to a scale of 0 to 100, with score of 100 being the best in health, and 0 the lowest. For SF-36, > 0 or <0 changes correspond respectively to an improvement (*blue lines*) or worsening (*orange lines*) of QOL. For NFTI-QOL, > 0 or <0 changes correspond respectively to a worsening (*orange lines*) or improvement (*blue lines*) of QOL in dizziness/balance (DIZZY), hearing, facial weakness (FACE), eyesight (SIGHT), walking (WALK),

life/family work (LIFE), pain, and anxiety/depression (MOOD) related questions. Each question in NFTI-QOL is scored from 0 to 3, for total score range from 0 to 24. Poorer QOL is represented with higher scores. NFTI-QOL score per domain is a mean score of all patients. (**c**) Distribution and percentage of patients in each tinnitus severity grade at baseline, 6 and 12 months of treatment. To increase the granularity of the responses, we used a score from 0 (extremely likely) to 10 (not at all). Total scores range from 0 to 250 and grades were adjusted from the original scale to catastrophic (0 to 42), severe (43 to 92), moderate (93 to 142), mild (143 to 192) and very mild (193 to 250). (**d**) Changes in tinnitus scores within the 3 subscales (functional, emotional and catastrophic) at 6 and 12 months of treatment normalized to each participant's baseline
